# Topology-controlled Pt atomic sites enhance electron utilization efficiency for NAD^+^ regeneration and alcohol detoxification

**DOI:** 10.1093/nsr/nwaf379

**Published:** 2025-09-11

**Authors:** Yinjun Tang, Pengcheng Qi, Yifei Chen, Jian Li, Wenxuan Jiang, Hongcheng Sun, Wenling Gu, Yao Sun, Chengzhou Zhu

**Affiliations:** State Key Laboratory of Green Pesticides, International Joint Research Center for Intelligent Biosensing Technology and Health, College of Chemistry, Central China Normal University, Wuhan 430079, China; Institute of Nano-Science and Technology, College of Physical Science and Technology, Central China Normal University, Wuhan 430079, China; State Key Laboratory of Green Pesticides, International Joint Research Center for Intelligent Biosensing Technology and Health, College of Chemistry, Central China Normal University, Wuhan 430079, China; State Key Laboratory of Green Pesticides, International Joint Research Center for Intelligent Biosensing Technology and Health, College of Chemistry, Central China Normal University, Wuhan 430079, China; State Key Laboratory of Green Pesticides, International Joint Research Center for Intelligent Biosensing Technology and Health, College of Chemistry, Central China Normal University, Wuhan 430079, China; College of Material Chemistry and Chemical Engineering, Key Laboratory of Organosilicon Chemistry and Material Technology, Ministry of Education, Hangzhou Normal University, Hangzhou 311121, China; State Key Laboratory of Green Pesticides, International Joint Research Center for Intelligent Biosensing Technology and Health, College of Chemistry, Central China Normal University, Wuhan 430079, China; State Key Laboratory of Green Pesticides, International Joint Research Center for Intelligent Biosensing Technology and Health, College of Chemistry, Central China Normal University, Wuhan 430079, China; State Key Laboratory of Green Pesticides, International Joint Research Center for Intelligent Biosensing Technology and Health, College of Chemistry, Central China Normal University, Wuhan 430079, China

**Keywords:** nanozymes, atomic sites, NADH oxidase mimics, coenzyme regeneration, alcoholism detoxification

## Abstract

Acute alcohol intoxication causes severe damage to the liver, nervous system and metabolic processes while inducing inflammatory responses. Current therapeutic strategies targeting ethanol metabolism through alcohol or aldehyde dehydrogenase activation face critical limitations of NADH accumulation and excessive reactive oxygen species (ROS) generation. Herein, by introducing a topology-controlled Pt atomic site into Co_3_O_4_ lattices, the geometric structure diversification of Pt was achieved for alcohol detoxification by coenzyme regeneration. In comparison with octahedral Pt_Oh_Co/O and pure Co_3_O_4_, Pt_Td_Co/O (atomic Pt sites within tetrahedral topology) exhibits 3.59- and 3.83-fold higher NOX-like activity, and preferentially catalyzes 4e⁻ oxygen reduction, improving electron utilization while minimizing ROS production. Mechanistic studies indicate that the introduction of Pt_Td_ sites improves substrate adsorption, enhances electron utilization efficiency and lowers the energy of reactions, thereby promoting alcohol degradation and reducing the release of ROS. This work on topological structure engineering offers new insights into designing high-performance biocatalysts for biological applications.

## Teaser

Due to the accumulation of NADH and ROS during alcohol metabolism, an effective solution is proposed for alcohol detoxification by coenzyme regeneration catalyzed by nanozymes with NADH oxidase-like activity by integrating single-atom Pt into Co_3_O_4_ lattices.

## INTRODUCTION

Although drinking has a long history as a social and recreational activity, alcoholism consistently poses major public health risks and has become a significant social issue globally. Alcohol, classified as a Group 1 carcinogen, is responsible for nearly three million deaths annually worldwide due to alcohol-related diseases [1–3]. Acute alcohol intoxication results from excessive drinking in a short period, causing a rapid spike in blood alcohol levels, which impairs brain function and can potentially be fatal [4–6]. Common treatments for acute alcoholism, such as gastrointestinal protection, gastric lavage and hemodialysis, have limited detoxification effects as they cannot directly metabolize alcohol [4]. Moreover, in clinical practice, drug detoxification primarily works by indirectly stimulating the activity of alcohol dehydrogenase (ADH) and aldehyde dehydrogenase (ALDH) [7,8]. However, excessive alcohol consumption causes a substantial accumulation of reduced nicotinamide adenine dinucleotides (NADH), which arises from the reduction of oxidized NADH (NAD^+^) acting as a coenzyme in the metabolic processes catalyzed by ADH and ALDH [9–11]. The disruption of the NADH/NAD^+^ ratio impairs the effective function of ADH and ALDH and disturbs redox balance, leading to increased reactive oxygen species (ROS) levels and triggering oxidative stress. Therefore, the rational design of alcohol detoxification drugs should effectively integrate coenzyme regeneration while minimizing ROS production.

Nanozymes with NADH oxidase (NOX)-like activity show great promise for alcohol detoxification by directly regulating the NADH/NAD^+^ ratio and oxidizing NADH *in situ*, effectively compensating for the absence of natural NOX in the body [12–17]. For nanozymes, the NOX-driven process comprises two distinct half-reactions: the dehydrogenation of NADH and the reduction of O_2_ (2e^−^ or 4e^−^ pathway) (Fig. S1 in the Supplementary data). First, the catalytic sites of nanozymes engage with the NADH molecule (the hydrogen atom at the C4 position) through hydrogen bonding or electrostatic interactions, thereby facilitating the transfer of protons and electrons from NADH to the nanozyme. The electrons extracted from NADH are then specifically utilized for O_2_ reduction [12]. Noble metal-based nanozymes are distinguished by their excellent catalytic activity, high chemical stability and versatile properties, positioning them as highly viable candidates for diverse applications, ranging from biosensing to therapeutic interventions [18–21]. However, noble metals such as NADH oxidase mimics remain largely unexplored, and systematic investigation into critical aspects such as catalytic mechanisms and application scenarios is significantly expected [17,22]. Their geometric structures, typically confined to thermodynamically stable close-packed modes like fcc and hcp, result in limited tunability of their binding sites and active sites for NADH oxidation in comparison with NOX [23–25]. In addition, unlike natural NOX that utilizes optimized active sites with precise cofactor–amino acid coordination, noble metal-based nanozymes exhibit compromised biocatalytic efficiency to activate substrate, which also inhibits the necessary electron transfer and proton transport processes from NADH to O_2_ [26–28]. Besides, the inefficient electron utilization results in a less effective 2e^−^ pathway for O_2_ reduction, producing H_2_O_2_ as a byproduct, which not only impedes ROS elimination but also restricts their potential for alcohol detoxification [28]. In this regard, rational design and synthesis of noble metal-based nanozymes to efficiently mimic NOX for alcohol metabolism remain a great challenge.

Herein, by integrating Pt into Co_3_O_4_ lattices, Pt atomic sites within a tetrahedral topology (Pt_Td_) and octahedral topology (Pt_Oh_) were synthesized for the *in situ* regeneration of NAD^+^ and alcohol detoxification. As a result, Pt_Td_Co/O exhibits a 3.59- and 3.83-fold increase in NOX-like activity in comparison with Pt_Oh_Co/O and Co_3_O_4_, while Pt nanoparticles do not exhibit NOX-like activity. Mechanistic studies reveal that Pt_Td_ sites, as the key active centers, oxidize NADH and transfer electrons to O_2_ to generate H_2_O by the 4e^−^ process, thus promoting their electron utilization efficiency and lowering reaction energy significantly. Moreover, Pt_Td_Co/O can effectively reduce the generation of ROS while promoting NAD^+^ regeneration. Benefiting from this strong ability, Pt_Td_Co/O shows striking therapeutic benefits by enhancing the metabolic activity of ADH and ALDH, thereby improving alcohol metabolism. This work opens a new way for designing noble metal-based nanozymes as NOX mimics for acute alcohol detoxification by fine-tuning their underlying topological structures at the atomic scale.

## RESULTS AND DISCUSSION

**Figure 1.**
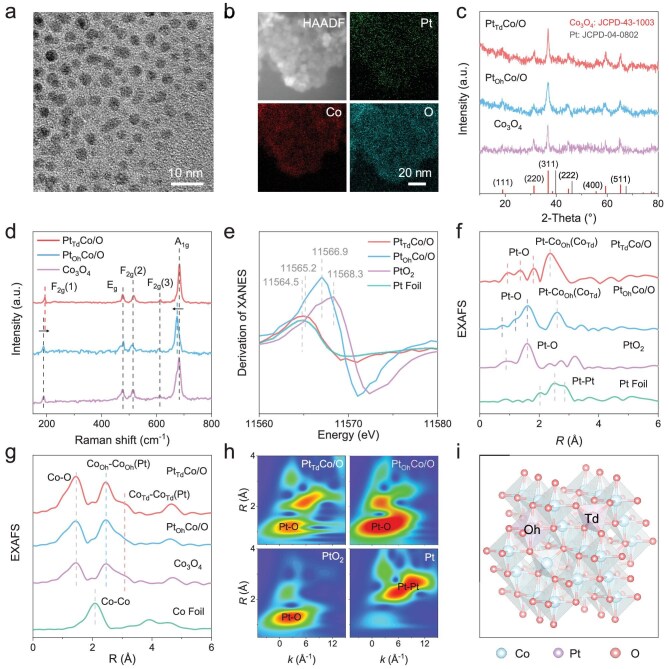
(a) TEM, (b) HAADF-STEM and the corresponding EDS mapping images of Pt_Td_Co/O. (c) XRD patterns, (d) Raman spectra, (e) first-order derivatives of the XANES spectra, (f) Pt L_3_-edge EXAFS spectra, (g) Co K-edge EXAFS spectra and (h) Pt L_3_-edge WTs of nanozymes. (i) Schematic representation of the structure of Co_3_O_4_ with topology-controlled Pt atomic sites.

The Co-based zeolitic imidazolate framework (ZIF-67) was used as the precursor, and potassium hexachloroplatinate(IV) (K_2_PtCl_6_) was incorporated into the host lattice under the controlled ion exchange and pyrolysis strategy (Fig. S2). The synthesis of Pt_Td_Co/O involved an ion exchange process conducted after pyrolysis, while an ion exchange process was carried out before pyrolysis to synthesize Pt_Oh_Co/O. Transmission electron microscopy (TEM) image (Fig. 1a) shows the nanoparticle structure of Pt_Td_Co/O. Similarly, Pt_Oh_Co/O and Co_3_O_4_ also exhibit the same morphology (Fig. S3). The high-resolution TEM (HRTEM) images of three nanozymes reveal lattice spacings of 0.24 nm, which correspond to the (311) crystal planes of Co_3_O_4_ (Fig. S4). The selected area electron diffraction (SAED) pattern shows that Pt_Td_Co/O and Pt_Oh_Co/O exhibit a similar crystalline structure to Co_3_O_4_ (Fig. S5). The energy-dispersive X-ray spectroscopy (EDS) images show a uniform distribution of Pt, Co and O elements in Pt_Td_Co/O (Fig. 1b) and Pt_Oh_Co/O (Fig. S6). The doping amounts of Pt in Pt_Td_Co/O and Pt_Oh_Co/O were measured by inductively coupled plasma mass spectrometry (ICP-MS), yielding similar values of 3.74 wt% and 3.48 wt%, respectively (Table S1). X-ray diffraction (XRD) was used to examine the crystalline structures of nanozymes. Pt_Td_Co/O and Pt_Oh_Co/O show similar characteristic peaks with Co_3_O_4_, indicating Pt was incorporated into the crystal lattice of Co without aggregating to form Pt nanoparticles (Fig. 1c). Raman spectroscopy is further used to characterize crystal structures and phase transition information of nanozymes (Fig. 1d). Raman spectroscopy reveals characteristic vibration peaks at 187.5 and 670.5 cm⁻^1^, corresponding to the Raman-active modes F_2g_(1) and A_1g_, which are associated with the tetrahedral (CoO_4_) and octahedral (CoO_6_) sites in Co_3_O_4_, respectively. It is noteworthy that the F_2g_(1) peak in Pt_Td_Co/O exhibits a shift, while the A_1__g_ peak remains unchanged, suggesting that the doped Pt occurs at the tetrahedral Co(II) sites (Co_Td_) [29]. On the contrary, the Pt sites are located at the octahedral Co(III) sites (Co_Oh_) in Pt_Oh_Co/O. Electron paramagnetic resonance (EPR) experiments confirm that the incorporation of Pt_Td_ increases the number of unpaired electrons in Pt_Td_Co/O compared with Pt_Oh_Co/O and Co_3_O_4_ (Fig. S7), which can be attributed to the lower oxidation state of Pt_Td_ causing more unpaired electrons in its d orbitals, whereas the higher oxidation state of Pt_Oh_ leads to greater electron delocalization and a more filled d orbital [30]. X-ray photoelectron spectroscopy (XPS) results show that the binding energy of the Co 2p peaks in Pt_Td_Co/O and Pt_Oh_Co/O shifts slightly positively by 0.6 eV in comparison with the Co_3_O_4_, indicating that electrons flow from Co to Pt sites (Fig. S8) [31]. As shown in Fig. S9, the d-band centers are positioned at 4.07, 4.46 and 4.19 eV for Pt_Td_Co/O, Pt_Oh_Co/O and Co_3_O_4_. The movement of the d-band center to the Fermi level of Pt_Td_Co/O indicates that the doped Pt_Td_ enhances its adsorption capacity to the substrate.

In addition, we further investigated the CO adsorption behavior of the nanozymes using a Fourier-transform infrared (FTIR) spectroscope to provide additional information about the dispersion and oxidation state of Pt. The characteristic absorption peaks at 2172.3 and 2120.6 cm^−1^, assigned to adsorbed CO on Co sites, are shown in Fig. S10 [32]. There is another peak of 2088.6 cm⁻^1^ in Pt_Td_Co/O, which corresponds to the linear adsorption of CO by atomic Pt_Td_ sites, further proving that Pt in this catalyst is monodisperse [33]. In contrast, the characteristic peak at this position is absent in Pt_Oh_Co/O, suggesting that Pt_Oh_ sites are not conducive to CO adsorption, while CO preferentially adsorbs on Co sites. Moreover, as shown in the K-edge X-ray absorption near-edge structure (XANES) of the nanozymes, the peak positions of Pt_Td_Co/O and Pt_Oh_Co/O closely resemble the peak profile of pure Co_3_O_4_, indicating that all three nanozymes share similar Co coordination structures (Fig. S11). The Pt L₃-edge derivation of the normalized XANES spectrum for Pt_Td_Co/O reveals that Pt exists in an oxidation state between 0 and +4, whereas for Pt_Oh_Co/O, the Pt oxidation state is closer to +4. This suggests that Pt in Pt_Td_Co/O favors tetrahedral coordination, while Pt with a higher oxidation state in Pt_Oh_Co/O is more likely to form octahedral coordination (Fig. 1e). The Pt L_3_-edge X-ray absorption fine structure (EXAFS) shows the characteristic interatomic distances for Pt–O and Pt–Co_Oh_(Co_Td_) of Pt_Td_Co/O and Pt_Oh_Co/O without Pt–Pt signals (Fig. 1f) [34,35]. The Co K-edge EXAFS reveals characteristic interatomic distances for Co–O, octahedral Co_Oh_–Co_Oh_(Pt) and tetrahedral Co_Td_–Co_Td_(Pt) in phases of Pt_Td_Co/O and Pt_Oh_Co/O (Fig. 1g) [36]. Significantly, the Co_Td_–Co_Td_(Pt) peak is more pronounced in Pt_Td_Co/O in comparison with Pt_Oh_Co/O and Co_3_O_4_. In addition, the Pt–O coordination number values based on the quantitative EXAFS fitting analysis are respectively 4 and 6 within Pt_Td_Co/O and Pt_Oh_Co/O (Table S2), further demonstrating that Pt in Pt_Td_Co/O is coordinated in a tetrahedral geometry, while Pt adopts an octahedral coordination within Pt_Oh_Co/O. Wavelet transforms (WTs) of Pt_Td_Co/O and Pt_Oh_Co/O present no apparent Pt–Pt signals, verifying that the Pt atoms are atomically dispersed in the lattice of Co_3_O_4_ (Fig. 1h and Fig. S12). As a result, the topological diversification of Pt atomic sites in the Co_3_O_4_ lattice was shown in Fig. 1i and Fig. S13.

**Figure 2.**
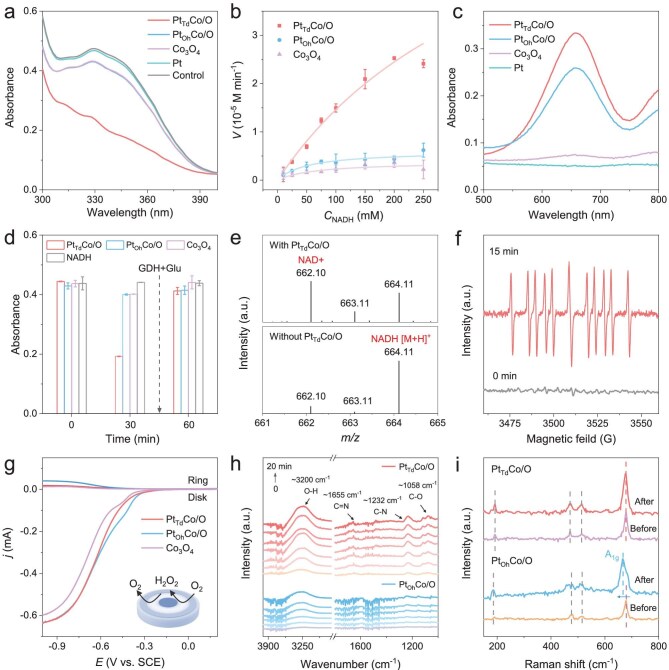
(a) Absorption spectra of the NADH oxidation reaction catalyzed by nanozymes. (b) The kinetic curve of nanozymes toward NADH. (c) The UV-Vis absorption spectra of the different solutions reacted for 20 min after adding TMB. (d) Absorption change at 340 nm before and after GDH (0.1 U) and glucose (10 mM) were added to the reaction mixture (nanozymes were removed). (e) Mass spectra of the NADH oxidation reaction with and without Pt_Td_Co/O. (f) ESR spectra of the spin adducts formed from CYPMPO in the reaction of NADH and Pt_Td_Co/O. (g) RRDE measurement of the selective oxygen reduction of nanozymes in the O_2_-saturated electrolyte (10 mM PBS, pH 7.4). Inset: schematic diagram of the RRDE used to test the selectivity of the reduction of the O_2_. (h) *In situ* ATR-FTIR spectra of nanozyme-induced NADH oxidation reactions. (i) Raman spectra of nanozymes before and after catalyzing the NADH oxidation.

To assess the NOX-like activity of nanozymes, we used NADH as a substrate, which exhibits a UV absorption peak at 340 nm [25]. Upon oxidation, the absorbance at this wavelength decreases, enabling quantitative measurement of the reaction rate. As a result, a significant decrease in the typical absorption of NADH at 340 nm was observed in the presence of Pt_Td_Co/O compared with Pt_Oh_Co/O and Co_3_O_4_ under the optimized test conditions (pH 7.4) (Fig. 2a and Fig. S14). To validate the superiority of Pt_Td_Co/O, nanozymes with lower-loaded Pt atomic sites (Pt_SA_Co/O) and Pt nanoparticles (Pt_NP_Co/O) integrated into Co_3_O_4_ were synthesized, and their enzyme-like activities were tested. The results show that Pt_Td_Co/O displays significantly higher specific NOX-like activity than Pt_SA_Co/O and Pt_NP_Co/O (Fig. S15). Similarly to natural enzymes, the NADH oxidation reaction catalyzed by nanozymes follows the Michaelis–Menten kinetic equation (Fig. 2b) [37]. The maximal reaction velocity (*V*_max_) of Pt_Td_Co/O is 3.37 and 3.82 times higher than those of Pt_Oh_Co/O and Co_3_O_4_. In addition, Pt_Td_Co/O has the lowest Michaelis–Menten constants (*K*_m_), demonstrating its better affinity for NADH. Notably, the kinetic constants of the Pt_Td_Co/O outperform those of most previously reported nanozymes (Fig. S16 and Table S3). The general chromogenic substrate 3,3′,5,5′-tetramethylbenzidine (TMB) was then used to evaluate the oxidase-like activity of nanozymes, and the results show that Pt_Td_Co/O exhibits the highest catalytic activity toward TMB, further demonstrating its ability to activate O_2_ (Fig. 2c). The NOX-like activity is markedly higher in an O_2_-saturated solution than in air and N_2_-saturated solutions, underscoring the essential role of O_2_ for NADH oxidation (Fig. S17). Nanozymes also demonstrate excellent stability and repeatability, highlighting their advantages over natural enzymes (Figs S18 and S19). To verify the products of NADH oxidation, glucose and glucose dehydrogenase were introduced into the system to confirm the generation of NAD^+^[25]. As shown in Fig. 2d, the recovery of NADH absorbance indicates that glucose dehydrogenase (GDH) successfully reduced NAD^+^, thereby confirming the presence of NAD^+^ within the system. This result is also supported by mass spectrometry analysis in Fig. 2e and ^1^H-NMR spectra in Fig. S20 [38]. After the oxidation of NADH, the mass spectra show new peaks at 662.10 *m/z* that can be assigned to NAD^+^. In addition, its chemical shift significantly increases, and a characteristic peak of NAD^+^ appears at 9.4 ppm. The intriguing catalytic properties prompt an in-depth investigation into the catalytic mechanism and reaction intermediates of nanozymes for NADH oxidation. As shown in Fig. 2f, EPR shows the presence of the intense signals of the NAD**⋅** intermediate, suggesting that Pt_Td_Co/O successfully extracted electrons from NADH for the subsequent O_2_ reduction. The rotating ring-disk electrode (RRDE) measurements were conducted to investigate the oxygen reduction process (Fig. 2g). The results indicate that both the disk and ring currents follow the same increasing trend, suggesting the reduction of O_2_. The electron transfer number of Pt_Td_Co/O and Co_3_O_4_ is 3.77, higher than the 3.40 of Pt_Oh_Co/O at the potential of −0.5 V, verifying that the introduction of Pt_Td_ favors the 4e^−^ reduction efficiency of O_2_ in comparison with Pt_Oh_ (Fig. S21a). The increased yield of H_2_O_2_ of Pt_Oh_Co/O also confirms this result (Fig. S21b). *In situ* attenuated-total-reflection FTIR (ATR-FTIR) spectroscopy was conducted to monitor the NADH oxidation process (Fig. 2h). The intensity of the O–H bond signal (∼3200 cm^−1^) is significantly increased over time, indicating the production of H_2_O. The oxidation of NADH coupled with O_2_ reduction leads to an increase in the infrared absorption peak intensity of the C=N (∼1655 cm^−1^) bond, while the formation of H_2_O results in enhanced O–H bond (∼3200 cm^−1^) stretching vibrations [39]. Besides, the formation of NAD^+^ alters the molecular conjugated system, increasing the polarity of the C–N (∼1232 cm^−1^) and C–O (∼1058 cm^−1^) bonds, which results in a greater change in dipole moment during vibration, and enhances the infrared absorption intensity [40]. All peaks are distinctly observed in the process catalyzed by Pt_Td_Co/O in comparison with Pt_Oh_Co/O, further verifying its superior catalytic activity. Raman spectra were further used to detect the structure change of nanozymes before and after catalyzing the NADH oxidation (Fig. 2i and Fig. S22). After the reaction, the A_1__g_ characteristic peaks of Pt_Oh_Co/O and Co_3_O_4_ shift, indicating a phase transition in their octahedral structure. In contrast, no peak shift is observed in Pt_Td_Co/O, confirming the stability of its structure and further highlighting the advantage of Pt substituting tetrahedral Co.

**Figure 3.**
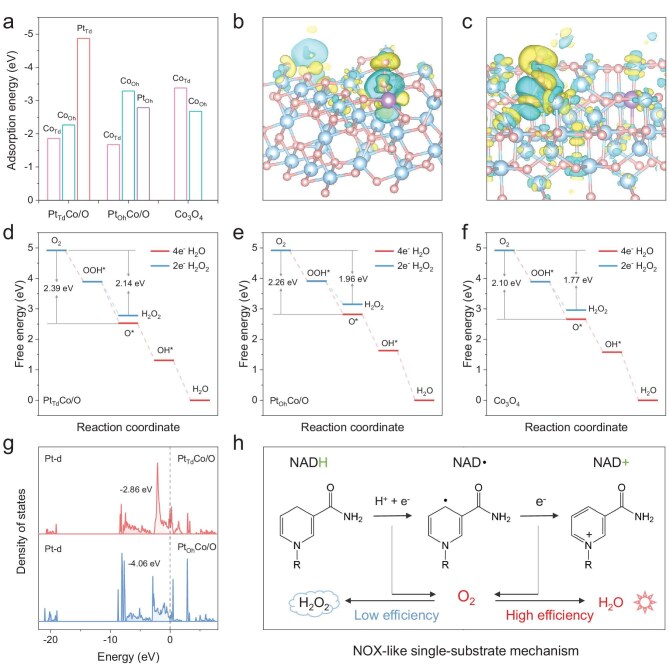
(a) The absorption energy of three nanozymes and their possible adsorption sites to O_2_. Calculated charge density differences to study the bonding interactions of (b) Pt_Td_Co/O and (c) Pt_Oh_Co/O among Pt (purple), Co (blue) and O (red) atoms, and the charge transfer (yellow/blue isosurfaces denote an increase/decrease of electron density); the purple, red and blue spheres represent Pt, O and Co atoms, respectively. The free energy diagrams of (d) Pt_Td_Co/O, (e) Pt_Oh_Co/O and (f) Co_3_O_4_ determined by the DFT studies. Projected DOS of (g) Pt-d of nanozymes. (h) Proposed mechanism of NADH oxidation and O_2_ reduction catalyzed by nanozymes.

Theoretical calculations were conducted to gain deeper insights into the mechanisms behind the improved NOX-like activity. The adsorption energy of nanozymes for O_2_ is summarized in Fig. 3a. The results verify that the Pt_Td_ sites in Pt_Td_Co/O demonstrate the highest affinity for O_2_ adsorption. Calculated charge density differences were then performed to study the electron transfer between O_2_ and nanozymes. Upon adsorption onto metal sites, noticeable electron migration occurs within the Pt–O bond of Pt_Td_Co/O and the Co–O bond of Pt_Oh_Co/O, leading to a reduction in electron density around Pt or Co atoms and an increase of electron density around O_2_ (Fig. 3b and c, Fig. S23). As shown in Fig. 3d–f, the energy changes for the 4e^−^ route of O_2_ reduction are respectively calculated to be −2.39, −2.26 and −2.10 eV of Pt_Td_Co/O, Pt_Oh_Co/O and Co_3_O_4_, which is lower than that of the 2e^−^ pathway (−2.14, −1.96 and −1.77 eV), confirming that all nanozymes prefer the 4e^−^ pathway. In addition, the lowest free energy change of Pt_Td_Co/O verifies its best catalytic capacity among nanozymes. The electronic densities of states (DOS) of these nanozymes were calculated to examine the adsorption of intermediates and electron transfer processes (Fig. 3g). The results reveal that the Pt d-band center of Pt_Td_Co/O (−2.86 eV) is close to the Fermi level compared to Pt_Oh_ sites in Pt_Oh_Co/O (−4.06 eV). This suggests that the Pt_Td_ site preferentially binds to O_2_, while simultaneously diminishing the ability of the surrounding Co site to interact with O₂. These results further verify the superiority of P_Td_ sites in Pt_Td_Co/O for NADH oxidation. These findings indicate that Pt_Td_Co/O facilitates the dehydrogenation of NADH into an NAD**·** intermediate, followed by the transfer of electrons from NADH to O_2_, which is subsequently reduced to H_2_O by the 4e^−^ pathway with higher efficiency (Fig. 3h).

**Figure 4.**
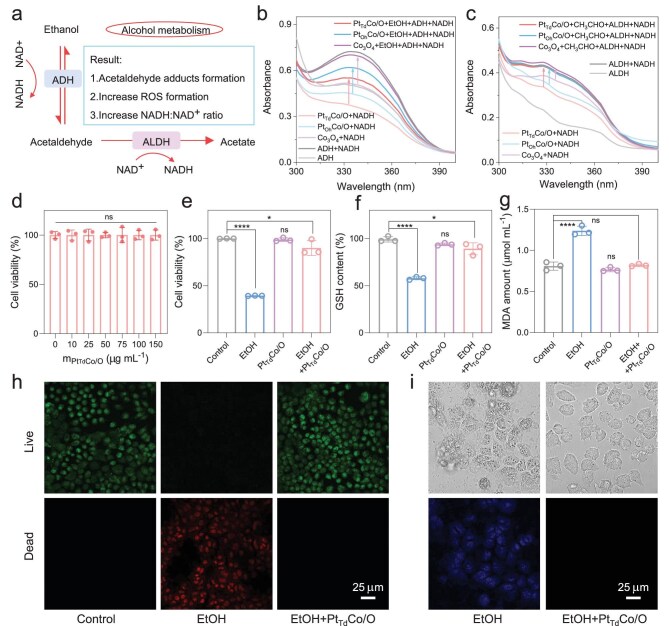
(a) Schematic illustration of the alcohol metabolic process. Absorption spectra of NADH oxidation and regeneration reaction catalyzed by nanozymes with (b) ADH or (c) ALDH. (d) Cell viability after treatment with different contents of Pt_Td_Co/O for AML12 cells. Experimental data for (e) relative cell activity, (f) GSH content and (g) MDA concentration of intracellular oxidative stress assessment of alcohol-stimulated AML12 hepatocytes in different treatment groups. (h) CLSM images of alcohol-stimulated AML12 hepatocytes in different treatment groups. (i) CLSM images of the morphology and the ROS generation in alcohol (EtOH)-stimulated AML12 hepatocytes. *P < 0.05; **P < 0.01; ***P < 0.001; ****P < 0.001; n.s., *P* > 0.05.

As illustrated in the alcohol metabolism diagram in Fig. 4a, excessive alcohol consumption into the human body results in the formation of acetaldehyde adducts, increased ROS production and an elevated NADH:NAD^+^ ratio. Therefore, we first explored whether nanozymes with NOX-like activity could solve the above issues caused by excessive consumption of alcohol. As shown in Fig. 4b, the characteristic peak of NADH can be recovered after the addition of ethanol and ADH. It suggests that nanozymes are capable of oxidizing NADH to generate NAD^+^, which then functions as a coenzyme for ADH in ethanol metabolism. Similarly, as shown in Fig. 4c, the absorbance at 340 nm increases upon the addition of acetaldehyde and ALDH, indicating that nanozymes can facilitate the metabolism of acetaldehyde. To evaluate the potential of nanozymes for alcohol detoxification, alpha mouse liver 12 (AML12) cells were used as models to investigate their cytoprotective effects. As shown in Fig. 4d, the good biocompatibility of the Pt_Td_Co/O was verified through MTT assays. The potential damage of alcohol to cells and the cytoprotective effect of Pt_Td_Co/O were then assessed by evaluating cell viability. Figure 4e shows that ethanol can cause significant cell damage, whereas the Pt_Td_Co/O helps maintain cell viability comparable to the control group [phosphate buffered saline (PBS) treatment]. To evaluate the intracellular oxidative stress status of alcohol-stimulated AML12 cells, the content of glutathione (GSH) and malondialdehyde (MDA) was measured and is shown in Fig. 4f and g [41]. Oxidative stress can deplete intracellular antioxidants, such as GSH, while also intensifying lipid peroxidation, leading to an increased production of MDA. As a result, ethanol can induce oxidative stress in cells, resulting in a decrease in GSH levels and an increase in MDA content. In contrast, the addition of Pt_Td_Co/O helps preserve cellular homeostasis in ethanol-treated cells. The confocal laser scanning microscopy (CLSM) images were then further used to determine cell status [42]. The live cell dye emits green fluorescence, whereas the dead cell dye exhibits red fluorescence. As shown in Fig. 4h and Fig. S24, when the cells are exposed to alcohol stimulation, there is a significant reduction in green fluorescence and an increase in red fluorescence, indicating that alcohol can induce cell apoptosis. In contrast, the addition of Pt_Td_Co/O maintains cell viability without inducing apoptosis, suggesting that nanozymes effectively metabolize alcohol and protect the cells. The bright-field images of the cells demonstrate that alcohol stimulation leads to significant damage to the cell morphology, while the addition of Pt_Td_Co/O helps preserve the cell morphology (Fig. 4i). Moreover, to confirm the generation of intracellular ROS through cascade nanocatalytic reactions, 2′,7′-dichlorofluorescein diacetate (DCFH-DA) was used as a probe. Upon ROS generation, DCFH-DA decomposes, producing strong blue fluorescence [11]. Alcohol can induce oxidative stress and increase ROS levels, enhancing fluorescence signals, while the addition of Pt_Td_Co/O reduces ROS levels and effectively maintains redox homeostasis. Mouse monocytic macrophage leukemia cell line (RAW264.7) cells, commonly used as inflammation models, also showed similar cell protection effects when treated with Pt_Td_Co/O (Fig. S25). Furthermore, EPR was used to detect the production of ROS using 5,5-dimethyl-1-pyrroline N-oxide as a trapping agent. As shown in Fig. S26, nanozymes cannot promote ROS generation and demonstrate a strong capacity to scavenge ROS that have already been produced. These findings highlight the potential of nanozymes for *in vivo* alcohol detoxification applications.

**Figure 5.**
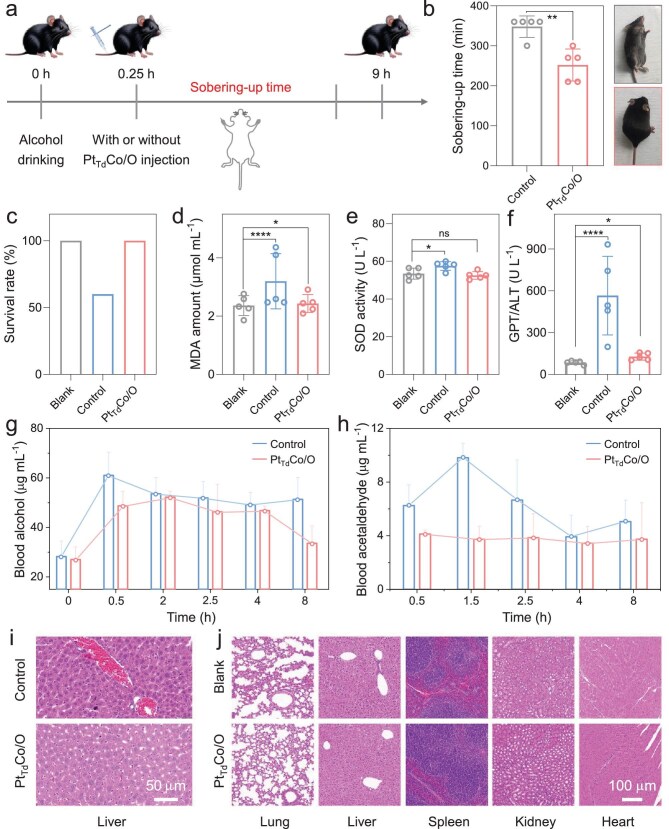
(a) Schematic of acute alcohol intoxication model construction (see Methods). Effect of different treatments (PBS, Pt_Td_Co/O) on (b) sobering-up time and (c) survival rate in C57BL/6 mice. (d) MDA amount, (e) SOD activity and (f) GPT/ALT amount in mice. Concentrations of (g) blood alcohol and (h) acetaldehyde in alcohol-intoxicated mice with different treatments. (i) Representative H&E-stained images of liver tissues from the two groups. (j) H&E slice images of different organs without and with Pt_Td_Co/O treatments. The tissues of mice were fixed with 4% paraformaldehyde, embedded in paraffin, and sliced. *P < 0.05; **P < 0.01; ***P < 0.001; ****P < 0.001; n.s., *P* > 0.05.

The effect of Pt_Td_Co/O on alcohol detoxification was assessed by constructing an acute mouse alcohol-poisoning model using C57BL/6 mice (Fig. 5a). The concentration of alcohol intragastric administration in the two groups was first optimized (Fig. S27). One group of mice was gavaged with alcohol as the control group, while the other group was gavaged with alcohol and injected with the Pt_Td_Co/O after 20 min as the experimental group. The results show that all alcohol-gavaged mice entered a sleep state due to alcohol intoxication, but the drug-injected mice woke up approximately 2 h earlier than the non-injected mice (Fig. 5b). Additionally, after about 12 h, only 60% of the mice survived without Pt_Td_Co/O injection, whereas the survival rate of the mice that received the drug injection remained at 100% (Fig. 5c). The liver is the main organ responsible for alcohol metabolism. It metabolizes ethanol through enzyme systems like ADH and ALDH, and further metabolizes acetaldehyde into non-toxic acetic acid, which is then either utilized by the body or excreted. By analyzing the MDA levels, superoxide dismutase (SOD) activity, and glutamic pyruvic transaminase (GPT)/ALT content in the blood of the mice, the stress response of the liver was assessed. As shown in Fig. 5d–f, while there is a slight increase in SOD activity, the MDA and ALT levels were markedly elevated in the ethanol-gavage group, indicating that alcohol gavage caused acute liver injury in the mice. In contrast, the group injected with the drug showed similar results to the control group, suggesting that the drug effectively alleviated the acute liver damage caused by alcohol. To investigate whether Pt_Td_Co/O can accelerate the metabolism of alcohol and acetaldehyde *in vivo*, we analyzed blood samples collected at different timepoints. After drug administration, ethanol levels in the blood decreased, and metabolism was accelerated (Fig. 5g). Moreover, there was no significant accumulation of acetaldehyde in the blood of the drug-treated group compared with the control group, which is vital for liver protection, since the accumulation of acetaldehyde is known to contribute to the development of liver cirrhosis and hepatocellular carcinoma (Fig. 5h). The hematoxylin and eosin (H&E) staining images of different organs without Pt_Td_Co/O treatment demonstrate that alcohol causes liver damage in mice (Fig. 5i). As indicated by the black arrows in Fig. S28a, the hepatocytes show signs of degeneration, characterized by cytoplasmic vacuolation and lighter staining. In contrast, the drug treatment significantly reduces alcohol-induced liver damage. As indicated by the black arrows, the hepatocyte structure appears normal, with no signs of degeneration, and the hepatic sinusoids show mild dilation and congestion (Fig. S28b). Physiological parameters, including body weight, food intake and water consumption, were monitored after Pt_Td_Co/O administration (Fig. S29). In addition, routine blood tests and liver/kidney function analyses were also performed (Table S4). All physiological indicators and biomarkers remained within normal ranges, demonstrating that Pt_Td_Co/O exhibits no significant systemic toxicity. Moreover, H&E staining revealed no significant tissue damage in the major organs after treatment with Pt_Td_Co/O (Fig. 5j), demonstrating its excellent biosafety [43]. These results highlight that nanozymes can effectively mitigate acute alcohol-induced liver injury by facilitating the metabolism of alcohol and acetaldehyde, while also reducing ROS and preserving the redox homeostasis of livers.

## CONCLUSION

In summary, to address acute alcohol-induced injury, we developed nanozymes with NOX-like activity by doping the topology-controlled Pt atomic sites into Co/O. The resultant Pt_Td_Co/O displays a 3.59- and 3.83-fold increase in NOX-like activity in comparison with Pt_Oh_Co/O and pristine Co_3_O_4_. Mechanistic studies confirm that the introduction of Pt atomic sites enhances the electron utilization efficiency extracted from NADH to favor the 4e^−^ oxygen reduction path. Moreover, Pt_Td_ sites are favorable for substrate activation, intermediate adsorption and electron transfer, thereby promoting the catalytic activity and selectivity of Pt_Td_Co/O. As a result, Pt_Td_Co/O effectively addresses the issues of elevated NADH levels, accumulated blood ethanol and acetaldehyde, and increased ROS following short-term high alcohol intake, thereby alleviating apoptosis and liver damage.

## MATERIALS AND METHODS

All animal experiments involved in this study were conducted in accordance with the Guide for the Care and Use of Laboratory Animals of Central China Normal University and were approved by the Ethics Committee (CCNU-IACUC-2023-001). The mice were raised with free access to standard feed and water in a 12 h dark-light cycle and the ambient conditions of room temperature (20–24°C), 50% ± 5% relative humidity. Detailed descriptions of materials and methods are available as supplementary data.

## Supplementary Material

nwaf379_Supplemental_File

## Data Availability

The authors declare that all data supporting the findings of this study are included within the article and its Supplementary data, as well as being available from the corresponding author upon reasonable request.
