# Supplementary material for: Topology-controlled Pt atomic sites enhance electron utilization efficiency for NAD+ regeneration and alcohol detoxification
Source: Natl Sci Rev. 2025 Sep 11;12(11):nwaf379. doi: 10.1093/nsr/nwaf379 (PMC12581896; doi:10.1093/nsr/nwaf379)
Supplement: nwaf379_Supplemental_File [file nwaf379_supplemental_file.pdf]

Supplementary information for

Topology-Controlled Pt Atomic Sites Enhance Electron Utilization Efficiency for NAD+ Regeneration and Alcohol Detoxification

Yinjun Tang,[1] Pengcheng Qi,[2] Yifei Chen,[1] Jian Li,[1] Wenxuan Jiang,[1] Hongcheng Sun,[3] Wenling Gu,[1] Yao Sun,[1] Chengzhou Zhu[1]*

[1] State Key Laboratory of Green Pesticide, International Joint Research Center for Intelligent Biosensing Technology and Health, College of Chemistry, Central China Normal University, Wuhan 430079 (P. R. China)

[2] Institute of Nano-Science and Technology, College of Physical Science and Technology, Central China Normal University, Wuhan 430079 (P. R. China)

[3] College of Material Chemistry and Chemical Engineering, Key Laboratory of Organosilicon Chemistry and Material Technology, Ministry of Education, Hangzhou Normal University, Hangzhou 311121 (P. R. China)

Contents

1. Experimental procedures
2. Supporting figures
3. Supporting tables
4. References

1.1 Instruments and materials

X-ray Diffraction (XRD) characterization was obtained by a D8 ADVANCE (Bruker, Germany). Transmission electron microscope (TEM) images were acquired from FEI Talos F200X. X-ray absorption near-edge structure (XANES) and extended X-ray absorption fine structure (EXAFS) experiments on a Fe K-edge were obtained from the 1W1B station in Beijing Synchrotron Radiation Facility (BSRF). The content of each element in the samples was determined by inductively coupled plasma optical emission spectrometry (ICP-OES) (iCAP 6300). X-ray photoelectron spectroscopy (XPS) measurements were performed by Escalab Xi+ (Thermo Fisher, United States). Electron paramagnetic resonance (EPR) spectra were obtained from a Bruker A300. *In situ* ATR-FTIR analysis was performed using a Nicolet iS50 FT-IR spectrometer (Thermo) equipped with a diamond internal reflection element (IRE) (refractive index n diamond = 2.4, incidence angle r = 42°). All enzyme kinetics data and UV-vis spectra were obtained by a multimode reader (Tecan Spark, Switzerland). The confocal laser scanning microscopy (CLSM) experiment utilized a Leica TCS SP8 microscope. All the absorbance and fluorescence spectra were obtained from a multimode reader (Tecan Spark, Switzerland).

Cobalt(II) nitrate hexahydrate ($Co(NO_3)_2$ $6H_2O$), potassium hexachloroplatinate(IV) (K_2PtCl_6), and hexadecyltrimethylammonium bromide (CTAB) were purchased from Aladdin Chemical Reagent Co., Ltd. 3, 3’, 5, 5’-tetramethylbenzidine (TMB) was bought from Sigma-Aldrich (St. Louis, MO, USA). Ethanol (EtOH) and hydrogen peroxide (H_2O_2) were purchased from Sinopharm (Shanghai, China). Reduced nicotinamide adenine dinucleotides (NADH) and glucose were purchased from Sigma-Aldrich. Alcohol dehydrogenase (ADH), alcohol dehydrogenase (ALDH), and glucose dehydrogenase (GDH) were ordered from Shanghai Yuanye Bio-Technology Co., Ltd. All cell culture and assay reagents were purchased from Elabscience Biotechnology Co., Ltd. All

chemicals were used as received without further purification. Ultrapure water was used throughout the experiments.

1.2 Computational methods

All calculations were performed using the Vienna ab initio simulation package (VASP) of first-principles calculations based on density functional theory. The core separation and valence electron interactions are described by projected added waves (PAW), and the local density is described using the generalized gradient approximation (GGA) based on the exchange-correlation energy PBE. Brillouin zone divisions were sampled using the Monkhorst-Pack method. ENCUT=400 eV was selected as the cutoff energy value for the calculation. The K points of dimensions 1*1*1-3*3*1 were generated by optimizing the convergence sampling in the calculation of the diffusion barrier and the mechanical strength, respectively. The crystal facet used for the slab construction during the simulation is $Pt_{Td}Co/O$ (311), $Pt_{Oh}Co/O$ (311), and Co_3O_4 (311).

1.3 Synthesis of ZIF-67

In a typical procedure, 580 mg of $Co(NO_3)_2 \cdot 6H_2O$ was dissolved in 20 mL of deionized (DI) water containing 30 mg of CTAB. Then, this solution was injected into 140 mL of aqueous solution with 9.08 g of 2-methylimidazole and stirred at room temperature for 60 min. The purple precipitate was collected by centrifugation and washed with ethanol five times.

1.4 Synthesis of nanozymes

The preparation of $Pt_{Td}Co/O$ involved an ion exchange process conducted after pyrolysis. First, Co_3O_4 was synthesized by pyrolyzing ZIF-67 nanocubes in the air at 300 ℃ for 4 hours. Then, 10 mL of an aqueous solution containing 5 mg of K_2PtCl_6 was added to 50 mL of an aqueous solution containing 100 mg of Co_3O_4 nanoparticles while stirring. Finally, 10 mL of an aqueous solution containing 10 mg of sodium borohydride was introduced to reduce K_2PtCl_6 and allow Pt species to be immobilized on the Co_3O_4 nanoparticle surface.

To prepare $Pt_{Oh}Co/O$, an ion exchange process was carried out before pyrolysis. Specifically, 100 mg of ZIF-67 nanocrystals were dispersed in 50 mL of deionized (DI) water. Subsequently, 5 mg of K_2PtCl_6 was dissolved in 10 mL of DI water and added dropwise to the ZIF-67 solution while stirring. After 3 hours, the reaction was quenched by centrifuging the mixture. The resulting precipitate was collected and washed twice with DI water and three times with ethanol. The $Pt_{Oh}Co/O$ was then obtained by drying the precipitate at 60 ℃ overnight in a vacuum oven, followed by pyrolysis in the air at 300 ℃ for 4 hours. $Pt_{SA}Co/O$ and $Pt_{NP}Co/O$ were synthesized by a similar method reported previously [1]. For the preparation of $Pt_{SA}Co/O$ and $Pt_{NP}Co/O$, a 10 mL solution of 1mg and 7.5 mg K_2PtCl_6 was added to 50 mL of 100 mg Co_3O_4 nanoparticle solution, followed by 10 mL of 20 mg sodium borohydride to reduce K_2PtCl_6.

1.5 The evaluation of NOX-like activities

For NADH oxidation, 1 mg mL^{-1} catalysts (0.2 mL) and 1 mM NADH (0.2 mL) were added into PBS buffer (10 mM, pH 7.4) and incubated for 30 mins. The supernatant was obtained by centrifuging. Then, the absorption spectra were recorded for further analysis. To determine the influence of different gas environments (N_2, O_2, and air), the corresponding atmospheric condition was maintained before and during measurements. The rapid color formation from the reaction can be conveniently measured on a spectrophotometer.

1.6 The evaluation of oxidase-like activity

The nanozymes (1 mg mL^{-1}, 10 μL) were introduced into the HAc-NaAc buffer (0.1 M, pH 3.0, 100 μL). Then, TMB (1 mM, 100 μL) was added to the above solution, and the absorbance values of the reaction solution were obtained by a multimode reader after 10 min.

1.7 Kinetic assay of NADH-like activities

1 mg/mL nanozymes (10 μL) and different concentrations of NADH (10, 25, 50, 75, 100, 150, 200, 250 mM) were added into the PBS buffer (10 mM, pH 7.4). The absorbance value at 340 nm was

recorded using a microplate reader for further analysis. The kinetic data can be obtained and fitted by the nonlinear regression of Michaelis-Menten following equation ($V=V_{max}[S]/(K_m+[S])$), where V is the initial velocity, [S] is the concentration of the substrate, K_m is the Michaelis constant, and V_{max} is the maximal reaction velocity.

1.8 EPR experiment

Nanozymes (1 mg mL^{-1}, 10 μL) and 10 mM H_2O_2 (20 μL) with and without Fe^{2+} were introduced into the PBS buffer (10 mM, pH 7.4) for 5 min. Then, DMPO (5 μL) was added to the above solution, and the EPR spectra of the reaction solution were monitored after incubation for 10 min.

1.9 Monitoring the NADH oxidation process of catalysts by in-situ FTIR

In total, 10 mg/mL nanozymes (5 μL) were first dropped on the diamond internal reflection element (IRE). The PBS buffer (10 mM, pH 7.4) was added, and the FTIR spectrum was recorded as a reference. Then, 50 mM NADH was further introduced, and the spectra were recorded every 2 min for further analysis.

1.10 Electrochemical measurement

An electrochemical workstation (CHI-760E) was employed for the electrochemical measurements at room temperature. A saturated calomel electrode acted as the reference electrode, and a Pt wire was used as the counter electrode. A glassy carbon electrode with a diameter of 3 mm was polished with 1.0 μm alumina powder and washed with Milli-Q water. Then, the working electrode was obtained by dropping 1 mg/mL of nanozymes solution (5 μL) onto the clean GCE and drying at 50 ℃. After that, 0.05 wt% Nafion (3 μL) was dropped onto the electrode surface and dried at 50 ℃.

1.11 Cell cultures.

Alpha Mouse Liver 12 (AML12) cells and Mouse Monocytic Macrophage Leukemia Cell Line (RAW264.7) were cultured in the special medium (Gibco, China) at 37 ℃ with 5% CO_2 in a constant temperature incubator.

1.12 In vitro cytotoxicity assay.

MTT assay was used to determine the cytotoxicity of nanozymes. Briefly, AML12 cells were seeded into 96-well plates at a density of 10^4 cells per well (200 μL) and were cultured at 37 °C with 5% CO_2 for 24 h. Then, $Pt_{Td}Co/O$ in different concentrations was added before another incubation for 24 h. Subsequently, 5 mg/mL of MTT (10 μL) was introduced and cultured for another 2 h. Finally, the media was removed and DMSO (150 μL) was added to each well, and cells' viability was obtained by a microplate reader. The cell viability was estimated according to the following equation: Cell viability (%) = $(A_{Treated}/A_{control}) \times 100\%$, where $A_{Treated}$ and $A_{Control}$ are the absorbance values with and without treatment of nanozymes, respectively.

1.13 In vitro alcohol detoxification of $Pt_{Td}Co/O$

For the alcohol detoxification tests, considering that cells cannot produce ADH and ALDH, AML12 cells were co-treated in a 96-well plate with alcohol (50 mM), NADH (10 mM), ADH (0.1 U/L), ALDH (0.1 U/L), and $Pt_{Td}Co/O$ (100 μg/mL) for 24 h. As a control, AML12 cells were adhesively incubated in a 96-well plate and then co-treated with alcohol (50 mM), NADH (10 mM), ADH (0.1 U/L), and ALDH (0.1 U/L) for 24 h. Another two control groups were also included: one without alcohol and one treated only with PBS. At the predetermined time, the cell viability was evaluated by MTT for the apoptosis assay. In addition, the concentrations of GSH and MDA in the lysate were determined using the corresponding detection kits to assess the intracellular oxidative stress of alcohol-stimulated AML12 cells after different treatments. For CLSM assays, confocal-plate-seeded AML12 cells were stained with Hoechst33342 /PI dye to check the live/dead situation after different treatments.

1.14 ROS detection in vitro

AML12 and RAW264.7 cells were used for the detection of intracellular ROS generation, and DCFH-DA was used to stain cells as a ROS fluorescent probe. Firstly, cells were seeded in 12-well

plates and allowed to adhere overnight. The culture media were then replaced by the special medium with different treatments. After incubation, 1 mL of special medium containing 10 μM DCFH-DA was added to each well and incubated for 20 min. All cells were washed using PBS, and then fluorescence images were obtained by CLSM.

1.15 Murine models

All animal experiments were approved by China's national regulations, and they received ethical and technical approval from the local animal welfare body. The mice (C57BL/6 mice, 6-7 weeks old) were raised with free access to standard feed and water in a 12 h dark-light cycle and the ambient conditions of room temperature (20-24 ℃), 50 ± 5% relative humidity.

1.16 Acute model

The mice with acute alcoholism were divided into two treatment groups, including PBS (positive control) and $Pt_{Td}Co/O$, while the group of normal healthy mice was used as the negative control. Each treatment group consisted of 5-10 mice. For in vivo acute alcoholism treatment, intoxicated mice received an intravenous injection of PBS or $Pt_{Td}Co/O$ (10 mg/kg) at 0.25 hours after alcohol administration. The blood concentrations of alcohol and aldehyde of intoxicated mice in different treatment groups were measured at different points in time within 8 h of treatment to analyze the metabolism of alcohol.

In addition, the righting reflex test was conducted to evaluate the recovery of consciousness in intoxicated mice following treatment. Eight hours after alcohol ingestion, the liver's stress response was evaluated by measuring malondialdehyde (MDA) levels, superoxide dismutase (SOD) activity, and glutamic pyruvic transaminase (GPT/ALT) content in the mice's blood.

1.17 Histological analysis

The main organs and hippocampus from the control group and treatment group were fixed in 10% formalin, embedded in paraffin wax, sectioned at 4 μm thickness, and stained with H&E for microscopic observation.

1.18 Statistical analysis

All data in this study were recorded as mean ± standard deviation (s.d.). A one-way analysis of variance was employed to analyze the significance of the difference, and the statistically significant P values are indicated in the legends.

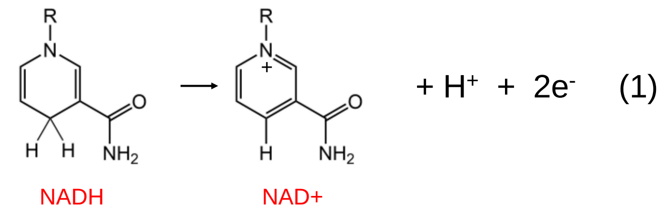

$$O_2 + 2H^+ + 2e^- \longrightarrow H_2O_2 \qquad (2)$$

$$O_2 + 4H^+ + 4e^- \longrightarrow H_2O \qquad (3)$$

Figure S1. Two individual half-reactions for NADH oxidation: (1) NADH and (2, 3) O_2 reduction.

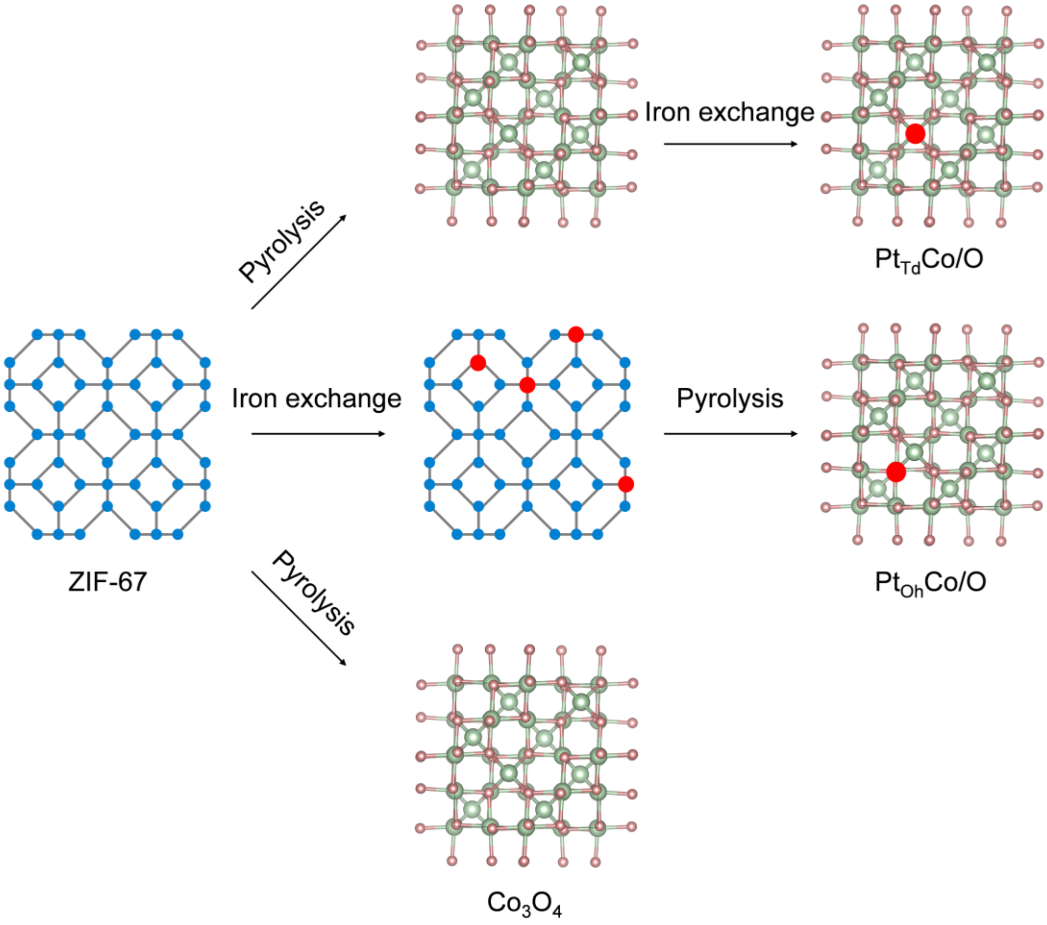

Figure S2. Schematic illustration for the preparation process of nanozymes.

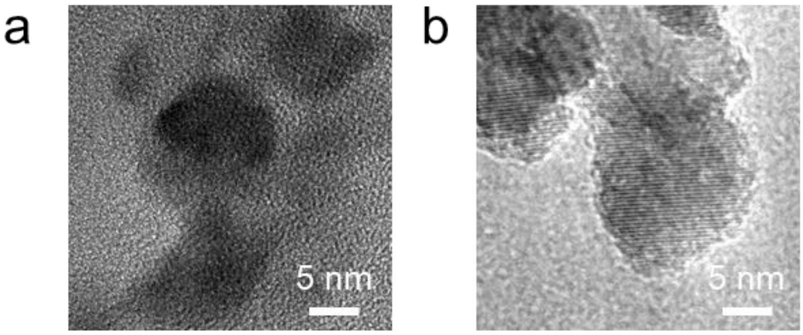

Figure S3. TEM images of (a) $Pt_{Oh}Co/O$ and (b) Co_3O_4.

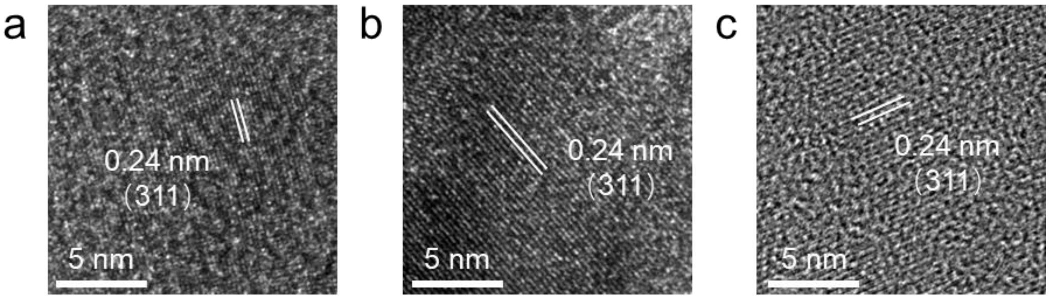

Figure S4. HRTEM images of (a) $Pt_{Td}Co/O$, (b) $Pt_{Oh}Co/O$ and (c) Co_3O_4.

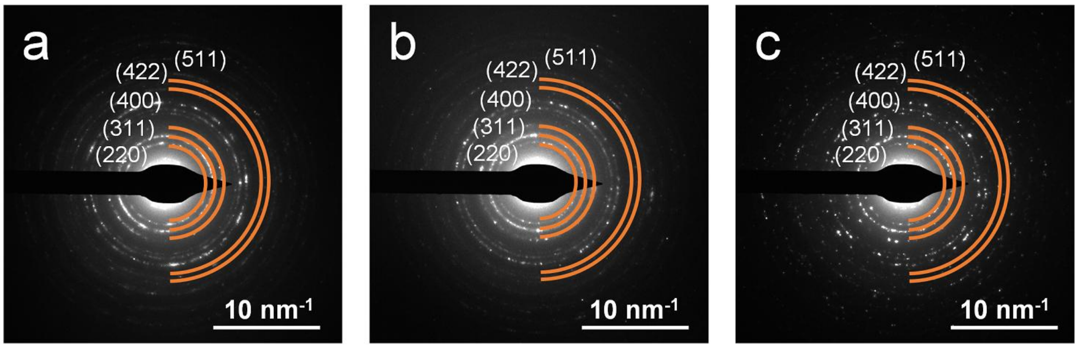

Figure S5. SAED patterns of (a) $Pt_{Td}Co/O$, (b) $Pt_{Oh}Co/O$ and (c) Co_3O_4.

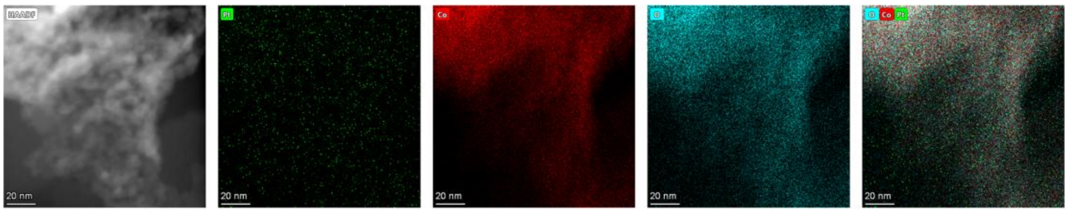

Figure S6. HAADF-STEM and the corresponding EDS mapping images of $Pt_{Oh}Co/O$.

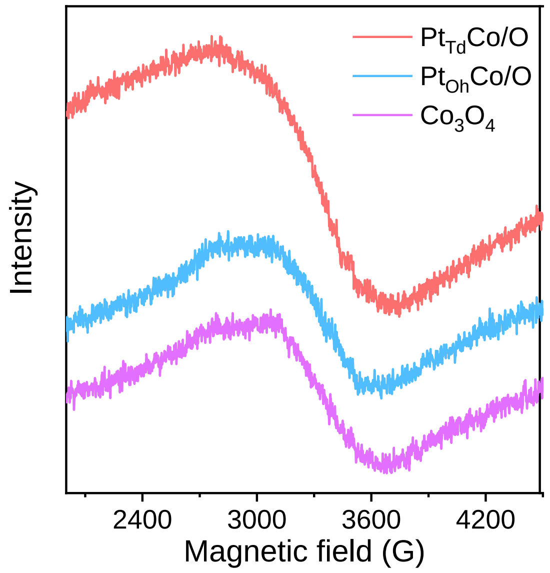

Figure S7. EPR signals of nanozymes.

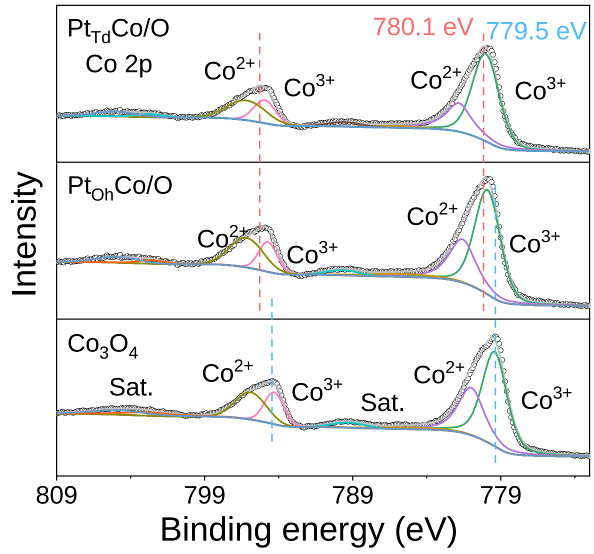

Figure S8. Co 2p XPS spectra of nanozymes.

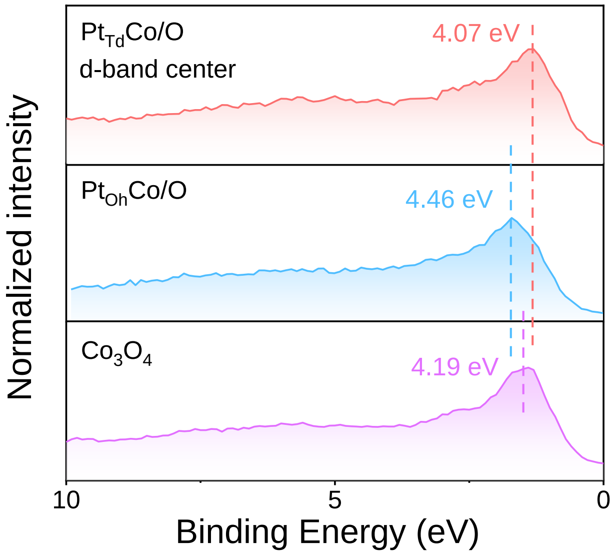

Figure S9. XPS valence band structures of nanozymes.

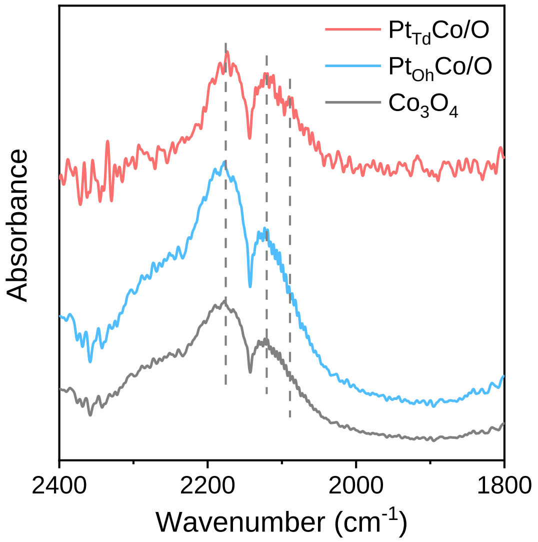

Figure S10. Fourier transform infrared studies of CO adsorption for nanozymes.

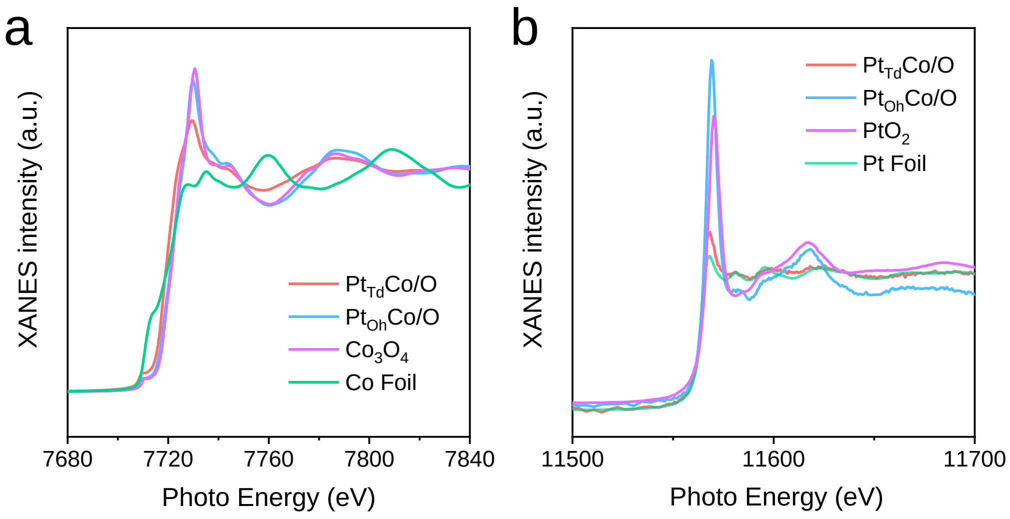

Figure S11. (a) Co K-edge and (b) Pt L_3-edge XANES spectra of catalysts.

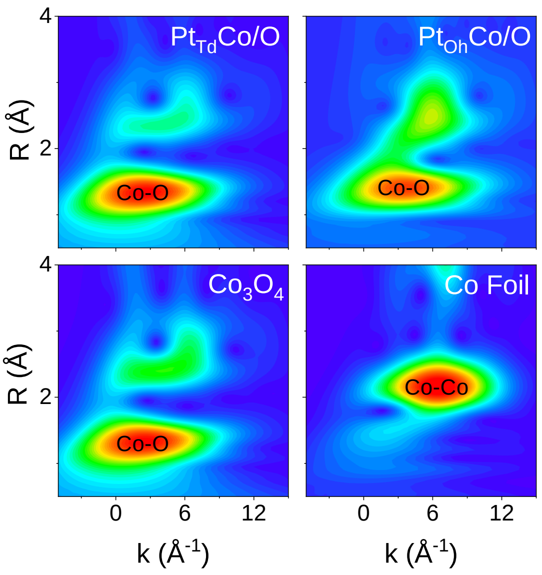

Figure S12. Co K-edge WTs of the resultant nanozymes.

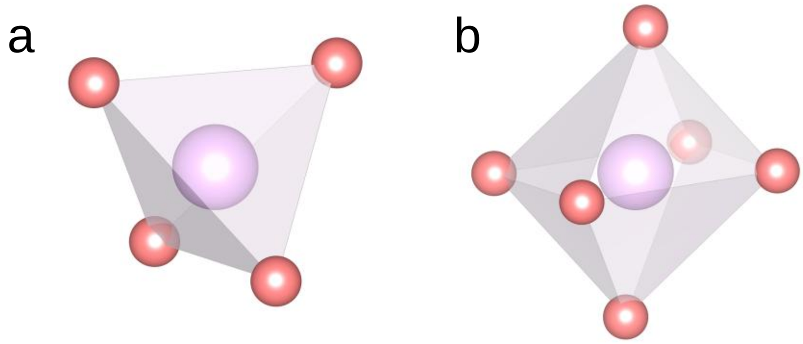

Figure S13. Schematic illustration of topological diversification of Pt single-atom sites in (a) $Pt_{Td}Co/O$ and (b) $Pt_{Oh}Co/O$. The red and blue spheres represent O and Co atoms, respectively.

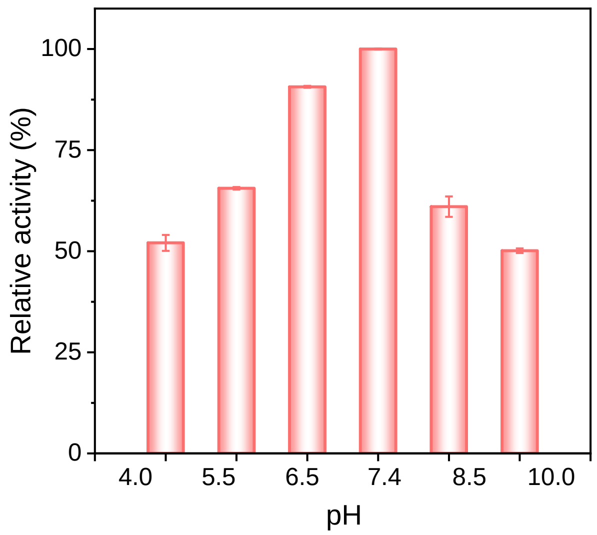

Figure S14. The NOX-like activities of $Pt_{Td}Co/O$ at different pH values.

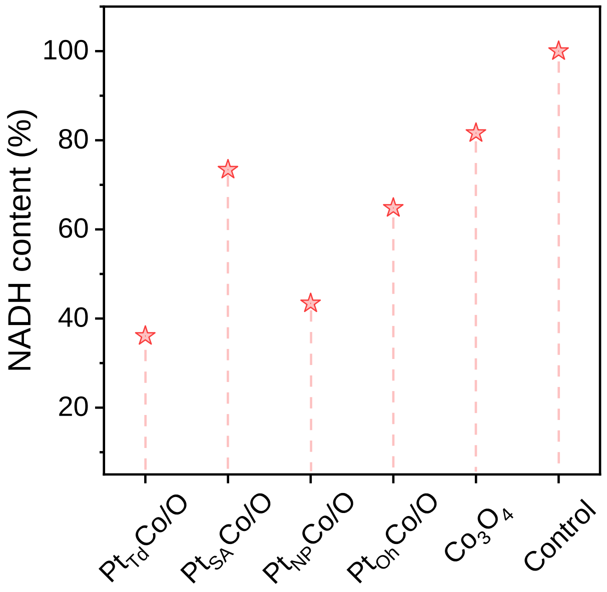

Figure S15. NADH content after NADH oxidation catalyzed by nanozymes.

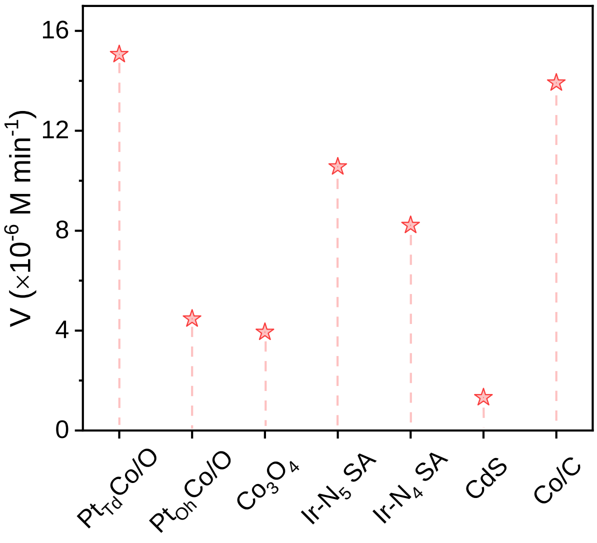

Figure S16. The V_{max} of nanozymes in this work and the reported literature for NADH oxidation.

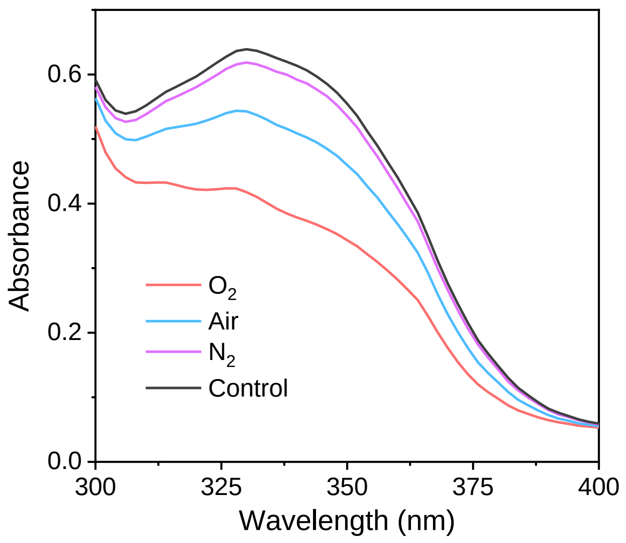

Figure S17. UV-vis absorption spectra of nanozyme-catalyzed NADH oxidation in the N_2-, air-, and O_2-saturated PBS solutions.

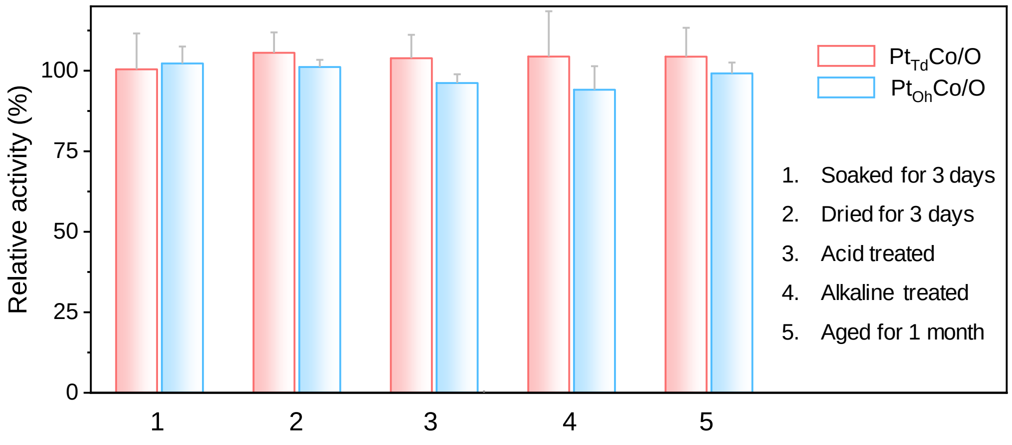

Figure S18. The relative NOX-like activity of different nanozymes.

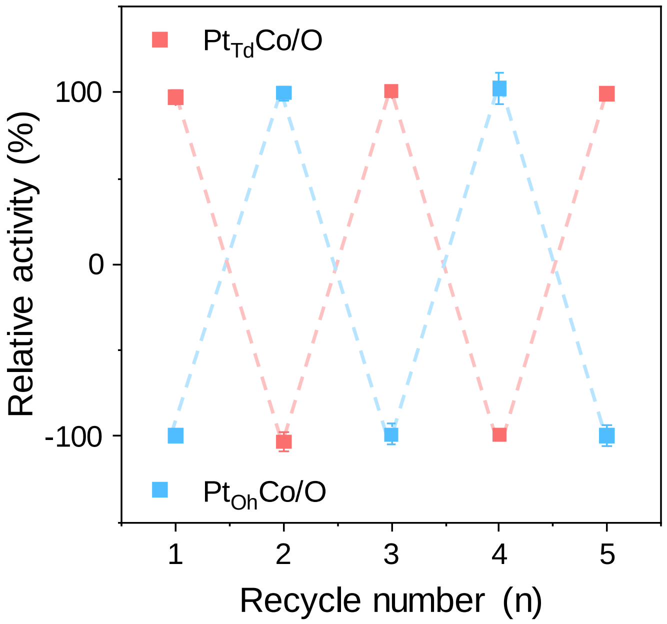

Figure S19. The relative NOX-like activity of different nanozymes.

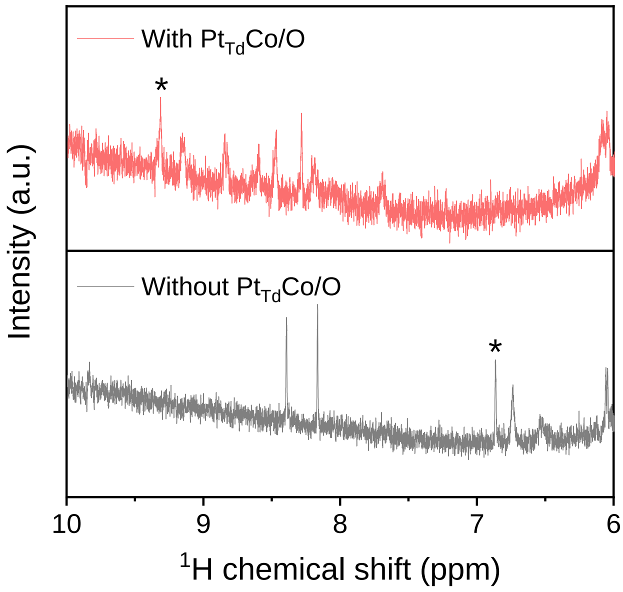

Figure S20. ^1H-NMR spectra with and without $Pt_{Td}Co/O$ for NADH oxidation.

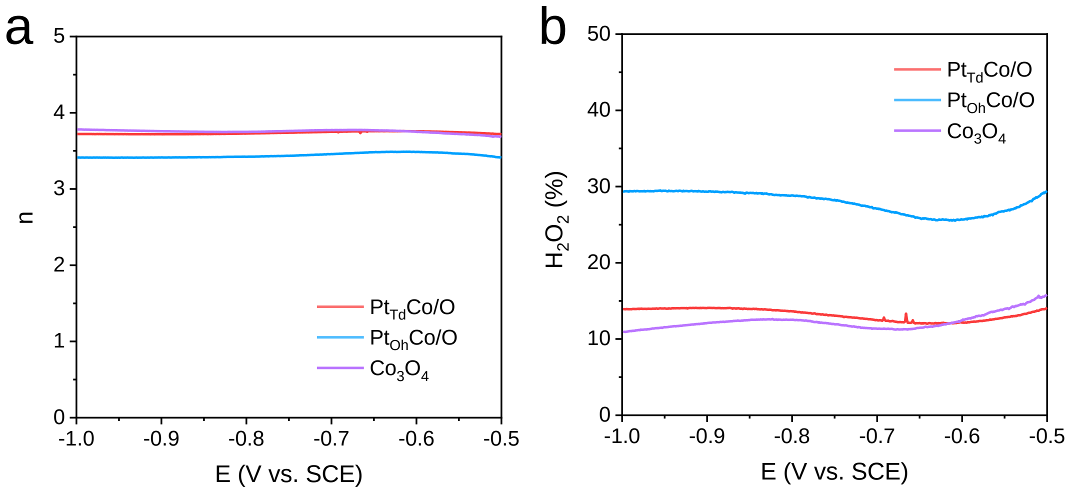

Figure S21. (a) Electron transfer number and (b) H_2O_2 selectivity versus the potential for nanozymes.

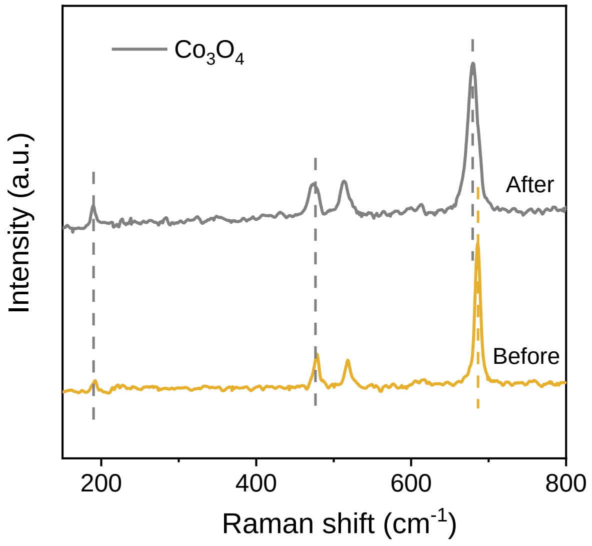

Figure S22. Raman spectra of Co_3O_4 before and after catalyzing the NADH oxidation.

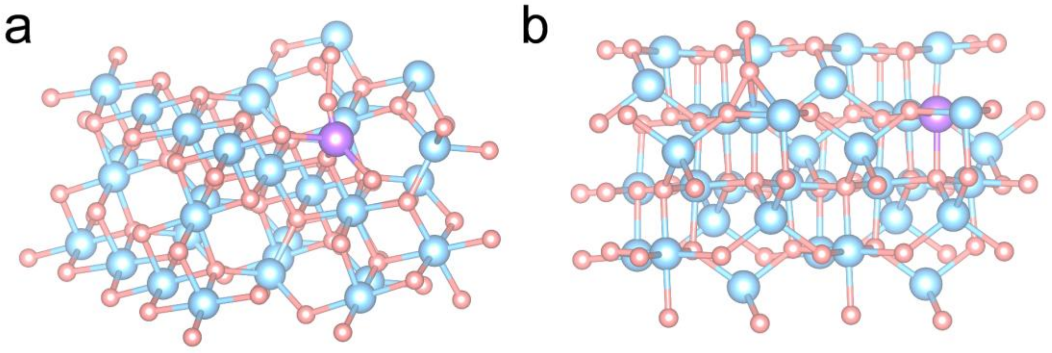

Figure S23. Calculated charge density differences study the bonding interactions of (a) $Pt_{Td}Co/O$ and (b) $Pt_{Oh}Co/O$ among Pt (purple), Co (blue), and O (red) atoms and the charge transfer, corresponding to Figure 3b and 3c.

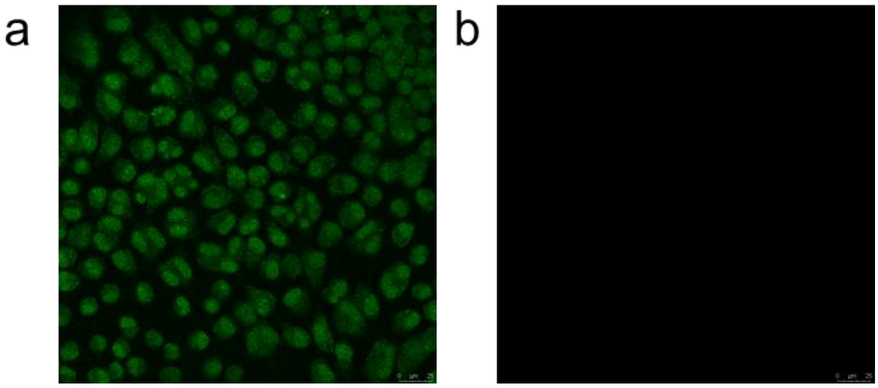

Figure S24. CLSM images of AML12 cells in $Pt_{Td}Co/O$ treatment groups without alcohol-stimulation, (a) the live cell dye emits green fluorescence, whereas (b) the dead cell dye exhibits red fluorescence.

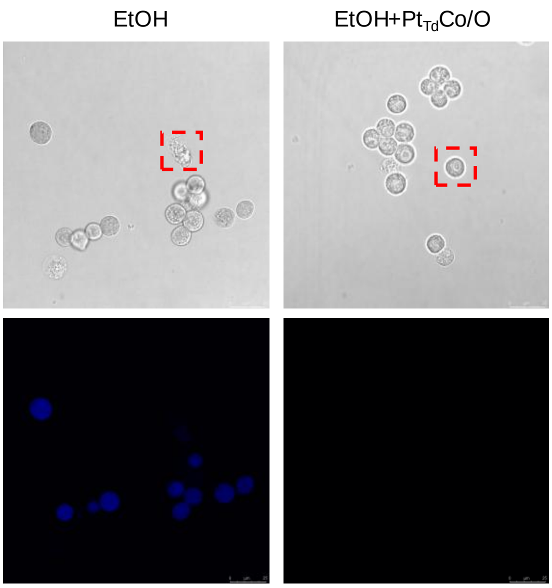

Figure S25. CLSM images of the ROS generation in alcohol-stimulated mouse monocytic macrophage leukemia cell line.

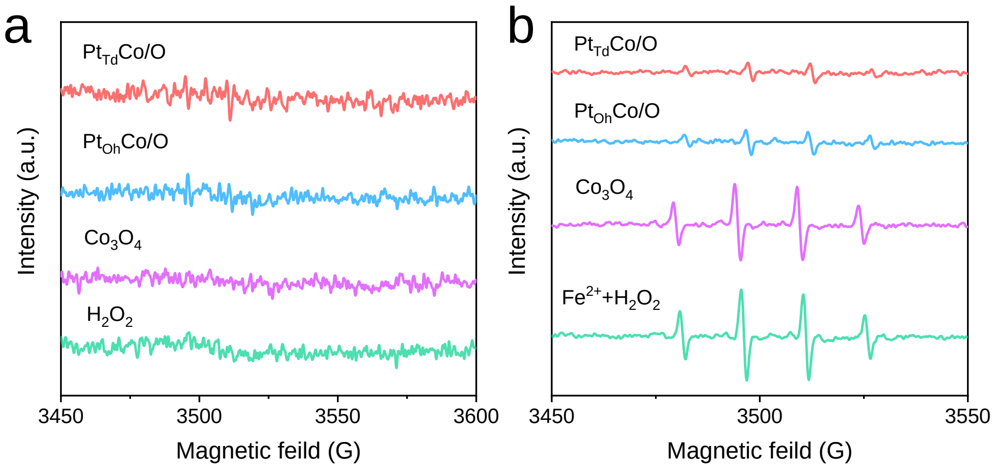

Figure S26. EPR spectra of nanozymes in the presence of (a) H_2O_2 (10 mM) and (b) Fe^{2+}+H_2O_2.

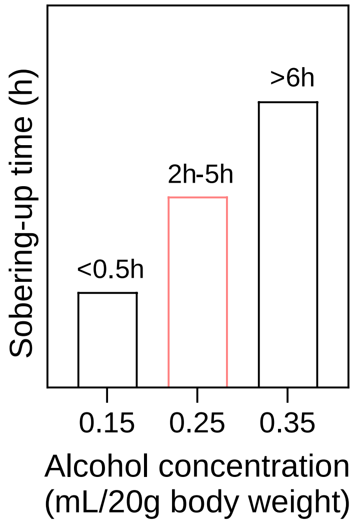

Figure S27. The effect of alcohol concentration on the sobering-up time of mice

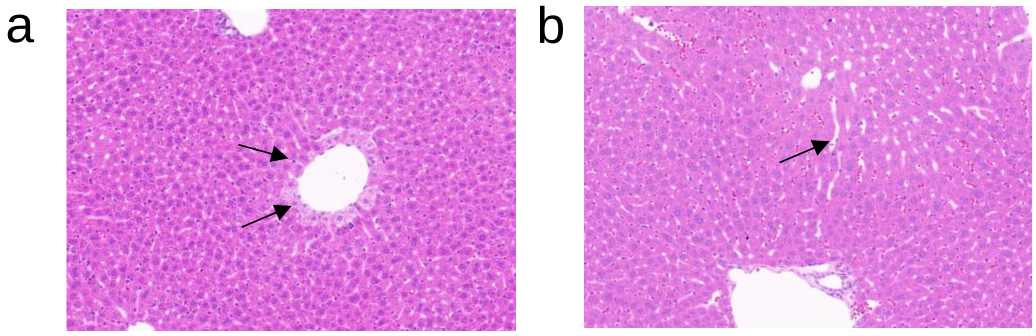

Figure S28. Representative H&E-stained images of liver tissues from groups (a) without and (b) with drug treatment.

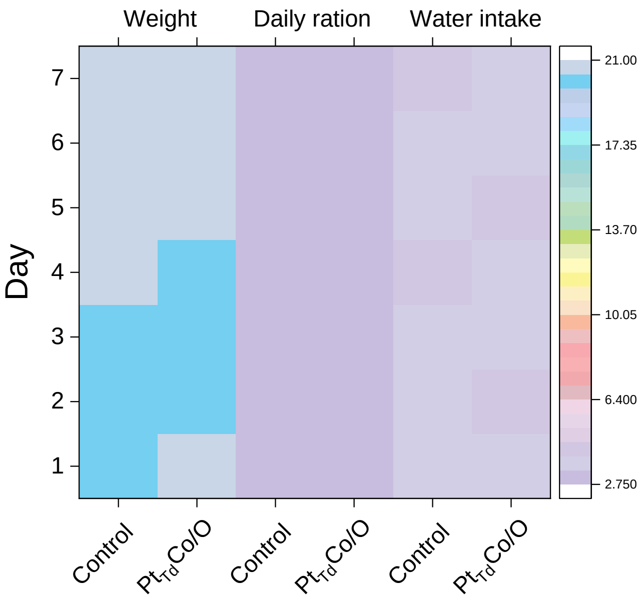

Figure S29. Temporal changes in physiological parameters of differentially treated mouse groups.

Table S1. The Co and Pt content of nanozymes detected by ICP-OES.

Catalysts	Co	Pt	Pt content (wt %)
$Pt_{Td}Co/O$	22.532	0.875	3.74
$Pt_{Oh}Co/O$	20.118	0.725	3.48

Table S2. EXAFS fitting parameters at the Pt L_3-edge for various samples.

Sample	Shell	CN	Bond length
$Pt_{Td}Co/O$	Pt-O	4.0	2.1
$Pt_{Oh}Co/O$	Pt-O	6.0	2.1

Table S3. Kinetic parameters of catalysts with NOX-like activity.

Catalysts	Substrate	V_{max} ($\times10^{-6}$ M min^{-1})	K_m (mM)
$Pt_{Td}Co/O$	NADH	15.06	69.27151
$Pt_{Oh}Co/O$	NADH	4.47	79.02872
Co_3O_4	NADH	3.94	145.21617
Ir-N_5 SA [2]	NADH	10.56	24.28
Ir-N_4 SA [2]	NADH	8.22	39.11
CdS [3]	NADH	1.32	418.81
Co/C [4]	NADH	13.92	70.50

Table S4. The blood biochemical and the blood routine examination indices under different treatments.

Indicator	Control group	Experimental group			Reference range
AST (U/L)	141.293	218.844	191.736	199.818	36.31-235.48
ALT (U/L)	45.675	38.322	58.442	49.258	10.06-96.47
ALP (U/L)	280.551	319.626	285.326	338.77	22.52-474.35
DBIL-F (umol/L)	12.078	16.304	19.500	19.634	0.45-33.89
T-BIL (umol/L)	15.88	21.448	19.692	16.306	6.09-53.06
CREA (umol/L)	45.611	68.708	84.04	49.04	10.91-85.09
UA (umol/L)	151.033	161.846	188.604	210.448	44.42-224.77
WBC (10^9/L)	6.5	6.6	8	7.6	0.8-10.6
LYM (10^9/L)	3.9	4	4.7	4.2	0.6-8.9
MON (10^9/L)	0.9	1	1.2	1.1	0.04-1.4
GRA (10^9/L)	1.7	1.6	2.1	2.3	0.23-3.6
LYM (%)	60	60.6	58.8	55.2	40-92
MON (%)	13.8	15.1	15	14.5	0.9-18
GRA (%)	26.1	24.2	26.2	30.2	6.5-50
HGB (g/L)	154.8	136.4	161.1	154.8	110-165
RBC (10^{12}/L)	8.99	8.06	9.21	8.54	6.5-11.5
HCT (%)	45.2	41.2	44.8	44.3	35-55
MCV (fL)	50.3	51.2	48.7	51.9	41-55
MCH (pg)	17.2	16.9	17.4	18.1	15.8-19
MCHC (g/L)	342	331	359	349	300-360
RDWCV (%)	12.9	13.1	14.8	13	12-19

AST: aspartate aminotransferase, ALT: alanine aminotransferase, ALP: alkaline phosphatase, DBIL-F: direct bilirubin, T-BIL: total bilirubin, CREA: creatinine, UA: uric acid, WBC: white blood cell count, LYM: lymphocyte count, MON: monocyte count, GRA: granulocyte count, LYM (%): lymphocyte percentage, MON (%): monocyte percentage, GRA (%): granulocyte percentage, HGB: hemoglobin concentration, RBC: red blood cell count, HCT (%): hematocrit percentage, MCV: mean corpuscular volume, MCH: mean corpuscular hemoglobin, MCHC: mean corpuscular hemoglobin concentration, RDW-CV (%): red cell distribution width.

REFERENCES

1 Shan J, Ye C, Chen S, *et al.* Short-range ordered iridium single atoms integrated into cobalt oxide spinel structure for highly efficient electrocatalytic water oxidation. *J Am Chem Soc*, 2021, **143**: 5201-5211.

2 Liu Y, Wang B, Zhu J, *et al.* Single-atom nanozyme with asymmetric electron distribution for tumor catalytic therapy by disrupting tumor redox and energy metabolism homeostasis. *Adv Mater*, 2023, **35**: 2208512.

3 Wang H, Chen J, Dong Q, *et al.* Cadmium sulfide as bifunctional mimics of NADH oxidase and cytochrome C reductase takes effect at physiological pH. *Nano Res*, 2022, **15**: 5256-5261.

4 Chen J, Zheng X, Zhang J, *et al.* Bubble-templated synthesis of nanocatalyst Co/C as NADH oxidase mimic. *Natl Sci Rev*, 2022, **9**: nwab186.
